# Developing a Theory-Driven Serious Game to Promote Prescription Opioid Safety Among Adolescents: Mixed Methods Study

**DOI:** 10.2196/18207

**Published:** 2020-07-03

**Authors:** Olufunmilola Abraham, Tanvee Thakur, Randall Brown

**Affiliations:** 1 Social and Administrative Sciences Division School of Pharmacy University of Wisconsin-Madison Madison, WI United States; 2 Department of Family Medicine and Community Health School of Medicine and Public Health University of Wisconsin-Madison Madison, WI United States

**Keywords:** opioid, medication adherence, adolescents, youth, video games, games, mobile phone, educational technology

## Abstract

**Background:**

Adolescents in North America are severely affected by the opioid crisis, yet there are limited educational resources for educating teens about prescription opioid safety and misuse. Empirical literature lacks evidence regarding teen education about prescription opioid safety through serious games and lacks conceptual models and frameworks to guide the process of game development for this purpose.

**Objective:**

This study aims to conceptualize and design a serious game prototype to teach teens about prescription opioid safety and propose a conceptual framework for developing a serious game to educate youth about safe and responsible use of prescription opioids.

**Methods:**

The initial steps of the project comprised of the formulation of an integrated conceptual framework that included factors from health behavior models and game development models. This was followed by the formal process of serious game development, which resulted in a game prototype. The assessment of the game prototype was done through group discussions, individual interviews, and questionnaires with adolescents following gameplay. Field notes were used to keep track of the responses from the group discussions. Content and thematic analyses were used to analyze field notes and responses to the open-ended questionnaire, which were then used to refine the game prototype.

**Results:**

A total of 10 playtests with over 319 adolescents and emerging young adults (AYAs) in community settings such as middle schools, high schools, and colleges were conducted by the project team between March and June 2019. The AYAs provided feedback on the initial game prototype using questionnaires administered through Qualtrics or in-person on paper. Preliminary feedback suggested that the teens found the game objectives, outcomes, and design appealing. Overall, the game was perceived as realistic, and learning outcomes seemed achievable. Suggestions for improvement included the need for additional direction on gameplay, clearer instructions, concise dialog, and reduced technical problems in the gameplay.

**Conclusions:**

We propose a conceptual framework for developing a serious game prototype to educate youth about prescription opioid safety. The project used a theory-driven conceptual framework for the development of a serious game targeting the prevention of adolescent opioid misuse and garnered preliminary feedback on the game to improve the quality of gameplay and the prototype. Feedback through informal assessments in community settings suggests that the youth and their families are interested in a game-based approach to learn about prescription opioid safety in homes and schools. The next steps include modifications to the game prototype based on feedback from the community, integration of learning analytics to track the in-game behaviors of players, and formal testing of the final prototype.

## Introduction

### Background

The misuse of prescription opioids and the associated negative health outcomes is a leading public health issue among teens in the United States [1]. Over the past 20 years, there has been an increase in the prescription and misuse of opioids among teens, as well as an increase in overdoses and overdose fatalities [1-3]. There is a significant risk for teens to experience an adverse drug event (such as sedation, physical dependence, or overdose) from either intentional or unintentional use of an opioid prescription [2]. In 2016, 11.8 million individuals aged 12 years or above living in the United States misused opioid medications [2]. Approximately 3.6% of adolescents aged between 12 and 17 years were reported to misuse opioids in 2016 [2]. Many teens and their family caregivers do not have a good understanding of the prescribed medications, particularly opioids [4]. Studies have shown that many individuals are not well educated about the side effects of opioid medications and the associated risks of improper storage. This lack of knowledge leads to adverse patient-centered events [4-9]. Hence, it is essential that teens receive developmentally appropriate education on opioid medication safety. Adolescent-focused education on the dangers of misusing or abusing prescription drugs, proper storage, and disposal of medications is increasingly recommended and is key in addressing inappropriate opioid use in this population [1].

Currently, there are few regulations or interventions to guide the education about opioids, particularly for teens [9]. Paper pamphlets are one of the few tools that exist; they tend to be theoretical and didactic in format and are neither patient-centered nor engaging. Educational materials that are intended to provide medication information to the youth and their families are at an 11th-grade reading level, which makes them difficult for all teens to comprehend [10-15]. Suboptimal educational materials and lack of awareness of the risks of opioid diversion and addiction in teens likely contribute significantly to the increase in nonmedical opioid use, misuse, addiction, overdoses, and deaths in this population [16-18].

Understanding and acting upon the existing medication information and instructions pose significant obstacles for almost half of the Americans [19,20]. Although a variety of opioid educational programs are available for adults, a digital tool designed to address the opioid medication safety knowledge gap in teens does not currently exist. Technology-based *serious games* are a novel method of delivering interactive health behavior education through skill-building exercises [21,22]. Serious games are digital tools that offer engagement activities through a responsive narrative to educate the participants through role-play and practicing skills. Unlike traditional video games, serious games act to convey meaningful information through interactive environments similar to real-life situations [23,24]. Serious games offer methods for increasing knowledge, delivering persuasive messages, changing behaviors, and influencing health outcomes among adolescents [1,25].

Although games for health offer promising positive patient outcomes [26-28], there is a dearth of research on the use of serious games to provide developmentally appropriate prescription opioid education for teens. A recent systematic review of serious games that incorporated medication usage identified only 12 serious games (targeting adolescents, young adults, and adults), with a majority aiming to increase adherence to specific medications or for a specific medical condition (such as cancer, diabetes, and asthma). Findings from the systematic review suggest an opportunity to use serious games to improve opioid medication safety and focus on preventing adverse drug events [29].

Behavioral theories, conceptual models for game development, and playtesting are known to improve the effectiveness of serious games [30,31]. There is limited evidence available about the use of specific theoretical frameworks to guide the game development process for medication safety–focused games for youth [29,32]. A few serious games about medication adherence and education have incorporated varying degrees of theoretical frameworks in their game design and assessment, but these games focus more on medication adherence than education about medications and effects [21,30-33]. Serious games that adopted theory in their design used small sample sizes for testing efficacy and effectiveness; hence, positive outcomes were demonstrated by only a few of these studies [30,34]. These studies applied either a gamification theory or the social behavioral theory to develop games incorporating medication usage as learning outcomes. To our knowledge, an integrated conceptual framework that includes concepts from both gamification and health behavior theories has not been developed and used to create a developmentally appropriate serious game for prescription opioid safety education for teens.

### Objectives

The *MEDSMAR_x_T: Adventures in PharmaCity* intervention was designed as a serious game to provide interactive education about prescription opioid risks and safety for adolescents. The goal of this game is to improve the knowledge, awareness, and self-efficacy teens have on the safe, appropriate, and responsible use of prescription opioid medications. Given the need for developing serious games to educate teens about prescription opioid safety and the lack of theory-driven conceptual models available in empirical literature to guide the process of game development, this project aimed to conceptualize and design a serious game prototype to educate adolescents about prescription opioid medication safety and propose a conceptual model for developing and assessing the use of the serious game in community-based settings. Community-based settings, in this context, include educational settings, such as schools, universities, and youth clubs, as well as health care settings in the community, such as hospitals, clinics, and pharmacies.

## Methods

### Conceptual Framework

**Figure 1:**
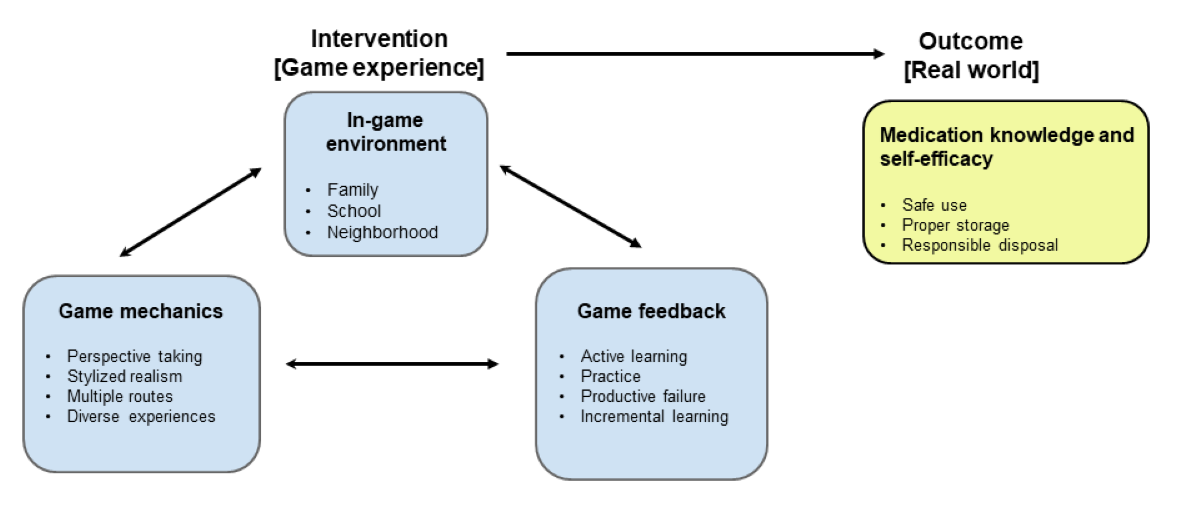
Serious game framework for improving medication safety in community settings.

A serious game behavior change framework for improving medication safety in community settings was developed (Figure 1) as the theoretical approach to guide this project. The proposed framework was based on behavioral and psychological interventions that incorporate a broad range of technologies, such as serious games, to positively change behaviors and cognitions related to health, mental health, and wellness [35]. The game intervention comprises 3 domains: the in-game environment, game mechanics, and game feedback, which are cyclically related to each other. The conceptual framework is derived from social behavioral and game development theories. Thus, the game experience is expected to lead to real-world outcomes (Figure 1).

The framework was informed by the principles of behavioral theories such as social cognitive theory and social learning theory. The principles from these models guided the selection of relevant constructs for the game experience and measurable learning outcomes such as medication self-efficacy [36,37]. The social cognitive theory posits that the combination of personal and environmental factors is a key influencer of behavior [38]. Each of these influencers was examined when creating this model, and game mechanisms that support them were identified. These mechanics were primarily informed by the learning principles from the foundational text by Gee, *What Video Games Have to Teach Us About Learning and Literacy*, and the literature on good instructional design practices [39-41]. The social learning theory proposes that individuals can acquire new behaviors by learning and imitating the behaviors of people with significant influences in their lives. In the case of adolescents, these include their parents, siblings, and friends. Thus, the in-game environment and the situations involved in the gameplay (such as events at home and school) were based on the principles of social learning and social cognitive theories [38]. The bidirectional interaction between personal and environmental factors and influential people who function as models for adolescents were considered significant locations and scenarios for the in-game environments (home or family, school, and neighborhood). The primary outcomes of *increased medication self-efficacy* in the conceptual model were informed by the social cognitive theory [38].

The behavioral theories were supported by the components of the behavioral intervention technology model, which integrates the principles of behavioral science, technology design, and engineering [42]. This theoretical framework defined both the conceptual and technological architecture of the game-based intervention by identifying the key concepts related to behavior change strategies such as education, design elements of technology, and characteristics of the interventions such as the needs, capabilities, and preferences of a user. Conceptually, the framework emphasizes the promotion or reduction of specific behaviors, the technology design characteristics, and the behavior change strategies.

Consistent with the theoretical dimension of the framework, the content of the serious game intervention will increase knowledge and teach adolescents essential skills to improve their self-efficacy in safe medication use, proper medication storage, and proper medication disposal, which are the expected outcomes of this intervention. The different terms used in the framework are given below.

The terms related to the game mechanics are as follows:

- Perspective taking: During gameplay, learners can take on identities to experience novel situations and roles, picture themselves in those settings, and build empathy toward the situation [39].
- Stylized realism: In game terminology, stylized realism involves using rich, authentically designed scenarios to support long-term learning [43].
- Multiple routes: Giving learners multiple options to explore empowers them and provides opportunities for replaying content. Showing different consequences of actions is powerful feedback [40,41].
- Diverse experiences: Simulations and games can provide a wide variety of experiences that are real enough to matter but still safe to explore [44].

The terms related to the game feedback are as follows:

- Active learning: In contrast to passive learning, active learning is a broad category that includes any instructional technique that engages the student in thoughtful, meaningful learning [45].
- Practice: Repeating attempts to recall and/or apply knowledge promotes long-term recall and transfer [46].
- Productive failure: This design strategy leverages short-term failure for long-term learning gains as learners come up with multiple attempts at solutions [47].
- Incremental learning: Learners have repeated attempts to produce a sense of challenge and develop learner persistence as well as repeated exposure to the learning material and relevant cues [43].

The framework suggests that the game-based intervention, which is a serious game experience, will have a direct effect on the learner’s knowledge and behaviors such as safe medication usage, proper medication storage and disposal, and educating peers or family members about opioid safety. Within the serious game experience, environmental factors where adolescents may interact are deliberately represented through game design and mechanics, such as support networks (peers), family (home environment), neighbors, and the school or city bus.

### Game Development Process

#### Creating a Multidisciplinary Team

The first step was to assemble an experienced project team with diverse and complementary expertise and experience in medication safety, adolescent health, opioid use, and game development. The project team comprised pharmacists, physicians, social behavioral health experts, student pharmacists, teens, game designers and developers, and researchers with expertise in improving medication use and health outcomes. The game developers were chosen based on their prior experience in developing serious games.

#### Knowing Your Audience

We assessed the baseline knowledge of the target population about prescription opioid medication misuse and safety as well as existing educational platforms preferred for learning about prescription opioid safety. This was conducted through formal and informal discussions with teens using focus groups, individual interviews, and questionnaires in the community. The target audience for this preliminary testing included middle school, high school, and college students.

#### Creating Learning Outcomes of the Game

The project team met weekly for 1 year (May 2018 through May 2019) to develop the first version of the serious game (MEDSMAR_x_T: Adventures in PharmaCity) prototype. The team meetings focused on identifying, discussing, and finalizing feasible and attainable learning goals for the game. The learning outcomes of the game were drawn from 8 principles of opioid safety agreed upon by the project team and informed by various opioid medication safety resources [48-50]. Players are expected to learn prescription opioid safety principles through gameplay and how to apply them in real-life situations. This includes safe usage, proper storage, and responsible disposal.

#### Applying the Conceptual Model

The MEDSMAR_x_T: Adventures in PharmaCity game aimed to improve knowledge, attitudes, and behaviors, and self-efficacy of adolescents regarding the safe and responsible use of prescription opioids. To meet this overarching goal, the project team created a conceptual framework comprising both gaming and health behavior theories. The conceptual framework entitled *a serious game behavior change framework for improving medication safety in community settings* (Figure 1) provided the theoretical underpinnings that guided this project. This framework was used to conceptualize learning objectives, gameplay outcomes, and game scenarios.

#### Creating a Game Playbook

The project team exchanged and shared ideas and knowledge about the behavioral and clinical health aspects and implications for game design and prototype development. A game playbook was created to serve as a guide for game design and development. It incorporated ideas from members of the multidisciplinary project team and was accepted by all on the team. This game playbook was critical to the team at different stages of prototype development to create and refine the content and to serve as source material for the preliminary informal testing of the game. The playbook also served as a reference resource for all team members for protocols and procedures.

#### Using an Iterative Feedback Process for Game Development

During weekly meetings, the team reviewed the progress of the game and provided feedback. Feedback was provided on domains (game environment and mechanics) of the conceptual model to ascertain that learning objectives were met, if the game was clinically relevant, and on the physical characteristics of the game (characters, aesthetics, and gameplay). The game was revised for 1 year, based on this continuous feedback and using an iterative process, until a playable game prototype was ready for gameplay and testing in community settings.

### Game Synopsis

**Figure 2:**
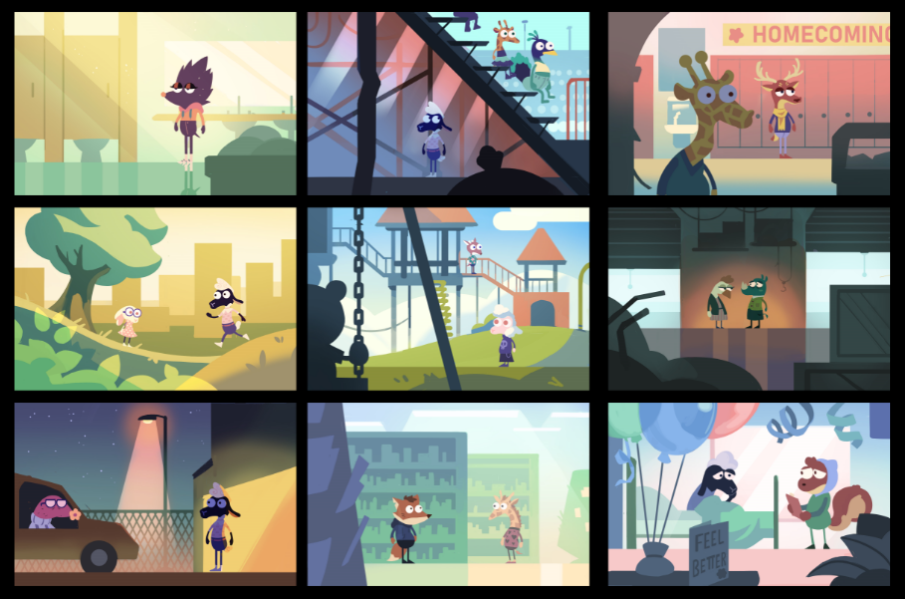
Anthropomorphized characters in the MEDSMAR_x_T game.

**Figure 3:**
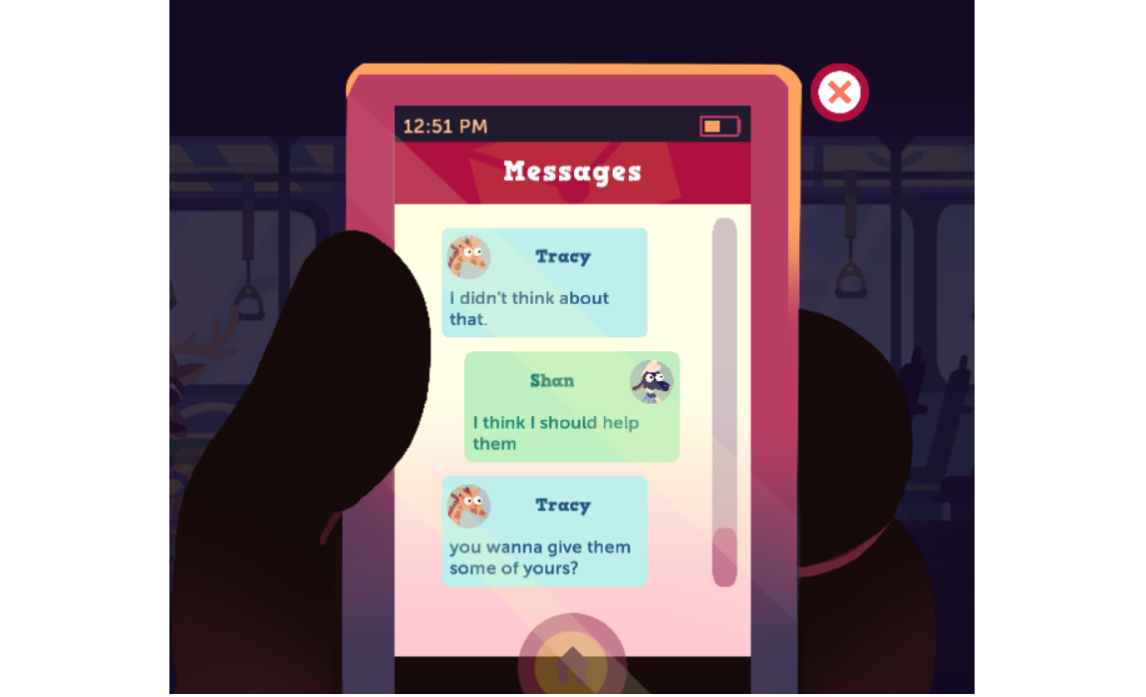
In-game cellphone dialogue.

The project team agreed on the use of an animated, animal-based character design for the MEDSMAR_x_T: Adventures in PharmaCity prototype game. Anthropomorphized characters were used, which may interest a diverse audience of gameplayers irrespective of gender, age, race, or ethnicity to easily identify with the game characters. The initial game prototype included animal characters, such as an anthropomorphized sheep as the main character (Figure 2). The anthropomorphic style of the game is reminiscent of currently popular games that target youth. Within the game, anthropomorphic characters interact within different environments and communicate between player and nonplayer characters using an in-game cell phone dialog mechanism (Figure 3). The use of such visually attractive images is supported in the literature as a means of connecting players with game synopsis, characters, and gameplay [51]. The project team emphasized using *animated stylized realism* that supported scenarios authentic to the real-world experiences of the youth. Rich, authentically designed scenarios support learning [52] and long-term retention of information and knowledge [53].

**Figure 4:**
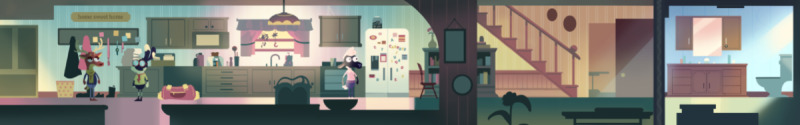
Home scenario in the game.

**Figure 5:**
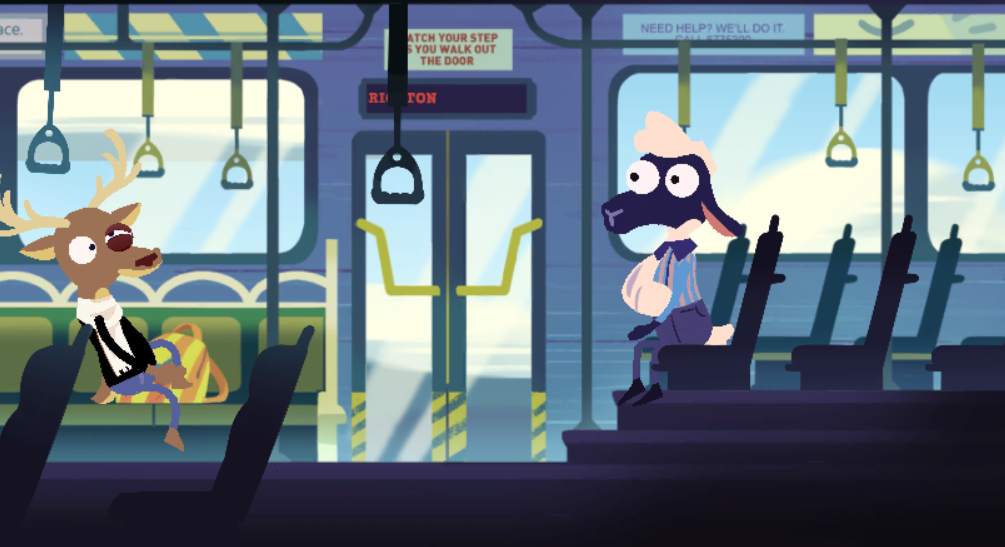
Bus scenario in the game.

**Figure 6:**
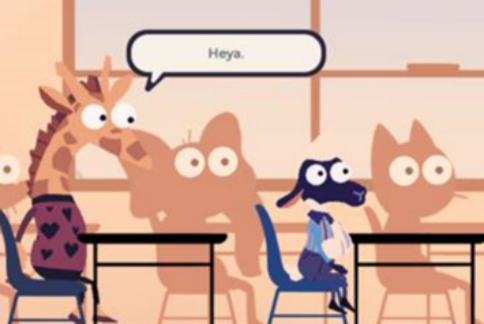
School classroom scenario in the game.

The game prototype design includes several locations that are reflective of community settings where youth might interact (ie, at home, in school, or on a bus). These situations were selected to highlight realistic settings where teens may engage in medication usage discussions and face ethical dilemmas related to the safe and responsible use of medicines. In each setting, players are asked to think from the lens of someone going through a situation involving opioid safety practices, thus implementing *perspective taking* in gameplay. Each situation highlights different challenges and requires decision making and critical thinking. *Active learning* is used here such that players in the game are faced with the positive and negative consequences of their choices as they explore options and develop strategies for navigating medication usage, storage, disposal, and related social pressures. Players could proceed to the next step only if they could successfully complete the previous level. Thus, the *practice* of opioid safety behaviors was implemented in the game in realistic scenarios such that players could also use them in the real world if needed. In the home scenarios, the player must discover how to safely store and dispose of prescribed opioids to avoid negative outcomes such as friends getting sick from finding and misusing these medications (Figure 4). In the bus scenario, the player is faced with a dilemma about sharing medications, accidental overdose, and naloxone use (Figure 5). At school (Figure 6), the player encounters another choice regarding prescription opioid misuse. All these different situations in the game provide *diverse experiences* for the gameplay.

**Figure 7:**
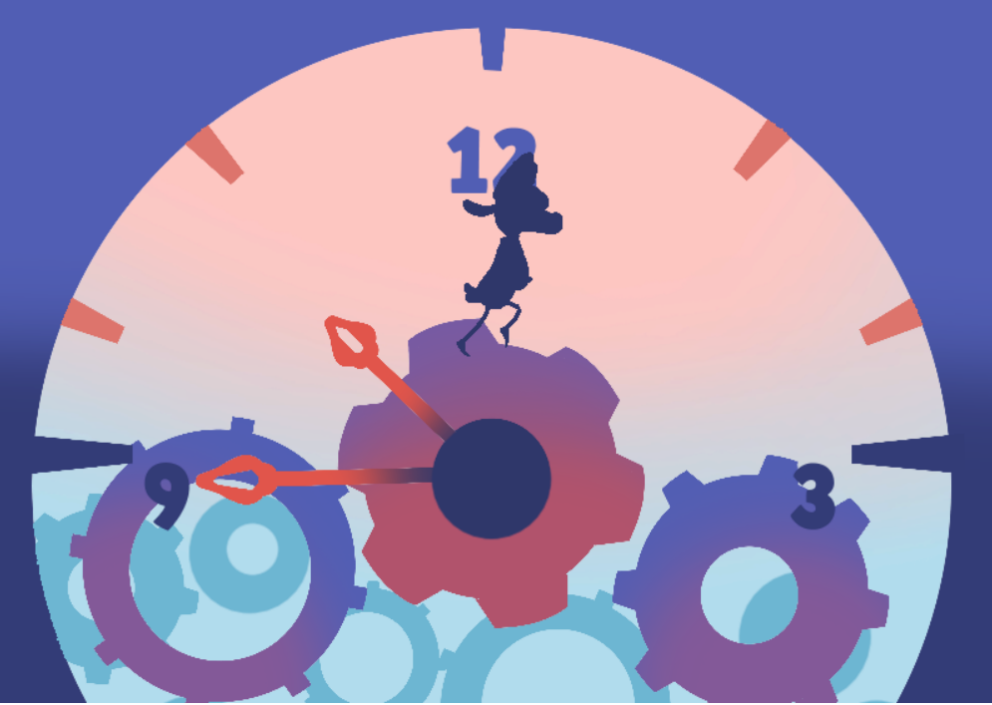
Repeating gameplay feature.

In these scenes, the player must choose how to respond, with options such as texting friends and family for advice. This provided an option of *multiple route*s for players where they could use one or more routes to decide and complete a level in the game. Players have an opportunity to *turn back the clock* and repeat scenes until they navigate them successfully (Figure 7), thereby learning from *productive failures* as well as successes. Repeated attempts also produce a sense of challenge and develop player persistence as well as repeated exposure to the learning material and relevant cues [41]. This *incremental learning* prompts learners to think back to the simulated situations when faced with analogous ones in the real world. This level of control and freedom is unique to interactive media tools such as serious games and promotes engagement and learning [39,41,43].

## Results

### Assessment and Evaluation of the Game Prototype

A total of 10 informal playtests with 319 adolescents and emerging young adults (AYAs) from middle schools, high schools, and colleges were conducted by the project team between March and June 2019. The participant sample was obtained by approaching school districts, universities, and youth clubs across the state of Wisconsin. Gameplay was conducted as a part of the activities in classroom sessions if allowed by the schools, universities, and youth clubs. The sites for preliminary testing and their characteristics are described in Table 1. The goal of the playtests was to obtain feedback to improve the quality of the game prototype related to game dynamics, design, and adaptability. Participants played the game individually and in groups. Feedback was obtained using individual questionnaires and through group discussions.

**Table 1:** Playtest sites and characteristics reported.

Site	Age (years)	Participants	Number of playtests
Middle school	11-13	Overall, 41.4% (132/319) students; 60.6% (80/132) male; race/ethnicity: 59.8% (79/132) white, 13.6% (18/132) Latino, 8.3% (11/132) African American, and 17.4% (23/132) Asian	6
High school	15-18	Overall, 4.4% (14/319) students; 7% (1/14) female; race/ethnicity: 79% (11/14) white and 7% (1/14) Asian	1
High school	15-17	Overall, 24.5% (78/319) students	3
Public library	14-19	Overall, 5.3% (17/319) high school students; 53% (9/17) male; race/ethnicity: 35% (6/17) white, 18% (3/17) Latino, 29% (5/17) African American, 6% (1/17) Native American, and 12% (2/17) Asian	1
Public library	12-15	Overall, 2.2% (7/319) students; 100% (7/7) male and white	1
Youth program	13-18	Not reported	3
University undergraduate students	20-21	Not reported	3
University undergraduate and professional students	17-24	Overall, 5.0% (16/319); race/ethnicity: 44% (7/16) white and 56% (9/16) Asian	2
University student pharmacists	24-26	Overall, 17.2% (55/319); race/ethnicity: 91% (50/55) white	1
University science fair	Not reported	Children and parents (not reported)	N/A^a^

^a^Not applicable.

### Community-Based Feedback

The questionnaire comprised open-ended questions about postgame opioid safety knowledge, experiences in gameplay, barriers and facilitators in gameplay, and opinions about the game. Group discussions after gameplay provided a mechanism for the project team to further understand gameplay barriers, facilitators, and overall feedback on the prototype. Responses were analyzed using content and thematic analysis involving open and axial coding. A total of 2 predominant themes emerged from the thematic analyses; the participants desired additional directions to guide gameplay and appreciated how the game demonstrated real-world scenarios and provided education that they could apply in real life.

The responses obtained from the questionnaire indicated that 78.4% (250/319) of the students perceived game scenarios as realistic. Participants reported the desire for more in-game hints and directions for gameplay. Participants reported that the game was appropriate for their age, whereas some high schoolers and college students thought that it was designed for younger populations. Overall, 94.6% (302/319) of the participants reported playing video games at home on a computer or phone every day. The scenarios, situations, and learning outcomes were reported to be realistic throughout the game. Suggestions for improvement from responses to the open-ended questionnaire included a need for additional background information, instructions, directions, or tutorials; expected actions; reducing the length of the dialog; increasing the speed of the dialog during gameplay; and updating the characters to reflect more human features. The group discussions shed light on the difficulties experienced by the players in navigating and transitioning between game scenarios and levels. The participants provided feedback on the game scenarios recommending that the safe and responsible medication management depicted in the game should align with the typical practices of families in both rural and urban settings.

## Discussion

### Principal Findings

The project used the serious game behavior change framework for improving medication safety in community settings to develop a serious game prototype, MEDSMAR_x_T: Adventures in PharmaCity. The game prototype is targeted for use among adolescents to prevent prescription opioid misuse. The creation of the initial game prototype involved a methodological approach that was multidisciplinary, iterative, and participatory and involved community stakeholders [54]. The project team garnered preliminary feedback from youth and suggestions to improve the quality of gameplay and the prototype design. Overall, the game was perceived as realistic, and learning outcomes seemed achievable. The game prototype was assessed using feedback from middle school, high school, and college students through informal group discussions and surveys. Half of the respondents perceived that the game was suited to their age, and the scenarios were realistic. Respondents reported barriers in gameplay, such as limited instructions and technical difficulty with some gameplay features. The result from this formative project will further improve the MEDSMAR_x_T: Adventures in PharmaCity game prototype. The feedback provides insights into the perceptions teens have about the game prototype. Although the game was perceived as realistic and the outcomes seemed achievable, teens also reported a need for iterations to the MEDSMAR_x_T: Adventures in PharmaCity prototype. Future directions and several limiting factors of game development are discussed below.

### Future Directions

The next step in this project is to polish and refine the MEDSMAR_x_T: Adventures in PharmaCity game based on feedback from AYAs, conduct formal pilot testing of the revised prototype, and create an in-game analytics infrastructure to accommodate data collection from a large number of game users. Building this infrastructure will help determine if the domains of the conceptual framework were successfully met and the avenues where the game requires revisions based on internal gameplay data. In-game data such as time to complete a level, time spent at various decision-making points, correct and incorrect answers, and progress within each level may enable meaningful analysis to determine the effectiveness of promoting prescription opioid safety among AYAs. Formal testing of the game’s effectiveness that incorporates in-game learning analytics data can provide a rich picture of a player’s gameplay experience and the potential impact of the game. Testing this game will begin with a randomized control trial that would include building learning analytics infrastructure into the game. Adapting for smartphones will be done simultaneously as well.

### Limiting Factors

#### Costs

Developing a serious game usually requires a much smaller budget than developing higher-quality commercial games. However, despite the availability of several game development and authoring tools, the complexity of developing a high-quality educational serious game is still beyond the reach of many developers. Creating a serious game incurs immense costs for game developers, especially for creating artistic assets such as graphics, characters, animation, dialog, and instructional design for players. The limited availability of funds prompted the project team to initiate the game design with key features, with future plans to incorporate in-depth gameplay infrastructure for data tracking for future iterations of the game.

#### Adoption and Deployment

Game deployment is the process of making the game available to the audience. Traditional PC games need to be installed in each machine before play. However, with current technology, providing a website to play the game is more common. The game must also be compatible with the operating system (ie, Windows or macOS) on the player’s computer. With institutional policies regarding data safety and monitoring, setting up a game in classrooms, hospitals, or pharmacies can be a daunting task for teachers, hospitals, and pharmacy staff. The current prominent use of mobile devices and the high prevalence of cell phone usage among teenagers makes the situation more practical as the operating systems are less variable compared with computer systems.

Game adoption is the process of actually using the serious game in the desired audience to make learning through the game possible. The audience for game adoption, in this case, is schools and health care settings, which often report a lack of adequate training on how to integrate the serious game in their teaching or consultations. Proactively identifying strategies to promote adoption in community settings would require collaboration with relevant stakeholders such as educators and health care providers to assess the best practices for dissemination and implementation in their respective practices. In addition, opportunities to creatively integrate the serious game into health and science classrooms and medication counseling sessions in health care settings are important considerations. The process of enabling effective adoption of the game requires more time, resources, and iterative playtesting, which involves integrating the game into real practice to reach the desired audience. Involving educators and health care professionals is critical to promote adoption and dissemination in community settings.

#### Building Learning Analytics Infrastructure

A major next step is to develop the learning analytics for the MEDSMAR_x_T: Adventures in PharmaCity game prototype. One study described learning analytics infrastructure as data collected in learning management systems (LMSs) or content management systems for improving teaching and learning [53]. One of the key aspects of learning analytics is tracking how many players interact with a web-based LMS by consulting access logs from the game server. An in-depth analysis of these logs can help identify behavior patterns that correlate with how the game is played. Incorporating the LMS features in game design such that it can be used for tracking gameplay requires financial resources and the expertise of game developers. In addition, this will enable data mining to extract and analyze these data. Involving all these components and experts in game design and development requires time, resources, and further playtesting.

#### Fast-Changing Technology

Rapidly changing and evolving technology is another challenge in disseminating this game to various audiences. Platforms for gameplay are constantly evolving, and adapting the game to the ever-evolving technology is both costly and time-consuming. This game prototype is currently developed for a computer but can easily be adapted to a smartphone in the future. Although challenging, keeping up with the constantly changing versions of smartphone and computer software would also be essential to overall game adoption and dissemination in the community.

#### Proposed Framework

The proposed framework is broad and has many categories. Further research and assessment are needed to understand the most important factors of the framework that directly affect gameplay, medication safety, and adolescent learning, which should be evaluated in quantitative trials [54].

### Conclusions

In summary, developing an audience-centered game is essential for its mainstream adoption. Therefore, it is crucial that the game content, learning outcomes, objectives, and game complexity incorporate feedback from the targeted AYA audience [55]. The project team sought to obtain initial feedback on the current game prototype from many AYAs in community settings. The feedback on gameplay, game design, and technical issues assisted the project team in fine-tuning the MEDSMAR_x_T: Adventures in PharmaCity game to ensure the creation of a consumer-centered prototype. The information learned through the extensive game prototype development process is foundational for improving and implementing MEDSMAR_x_T: Adventures in PharmaCity on a larger scale in community settings.

This formative project describes the development process of MEDSMAR_x_T: Adventures in PharmaCity. We propose a conceptual model entitled the *serious game behavior change framework for improving medication safety in community settings* grounded in social behavior and game development theories. The framework may be used for developing serious games to educate youth about prescription opioid safety**.** The project utilized a theory-driven conceptual framework for the development of a serious game targeting adolescent opioid misuse prevention and garnered preliminary feedback on the game to improve the quality of gameplay and the prototype. Feedback through informal assessments in community settings suggest that the youth and their families were interested in a game-based approach to learning about prescription opioid safety in homes and schools. The next steps include modifications to the game prototype based on community-based feedback, integrating learning analytics to track players’ in-game behaviors, and formal testing of the final prototype. The future directions constitute making this game prototype more consumer-centered and integrating the learning analytics framework in the game to track the progress of players and gaming behaviors in gameplay.

## Abbreviations

AYA: adolescent and emerging young adult
LMS: learning management system
NIH: National Institutes of Health
